# Involving supermarkets in health promotion interventions in the Danish Project SoL. A practice-oriented qualitative study on the engagement of supermarket staff and managers

**DOI:** 10.1186/s12889-023-15501-5

**Published:** 2023-04-18

**Authors:** Lise L. Winkler, Ulla Toft, Charlotte Glümer, Paul Bloch, Tine Buch-Andersen, Ulla Christensen

**Affiliations:** 1 Center for Clinical Research and Prevention, Copenhagen University Hospital - Bispebjerg and Frederiksberg, Nordre Fasanvej 57, Frederiksberg, 2000 Denmark; 2 Center for Diabetes, Vesterbrogade 121, 3rd floor, København V, 1620 Denmark; 3 Health Promotion Research, Steno Diabetes Center Copenhagen, Borgmester Ib Juuls Vej 83, Herlev, 2730 Denmark; 4 Section of Social Medicine, Department of Public Health, University of Copenhagen, Gothersgade 160, København K, 1123 Denmark

**Keywords:** Supermarkets, Real-life intervention, Community-based health promotion, Practice theory, Qualitative research

## Abstract

**Background:**

Supermarkets have been suggested as relevant settings for environmental and educational initiatives encouraging healthier shopping and eating decisions, but in the literature, limited attention has been paid to the context, perspectives, and everyday practices of supermarket staff. The aim of this study was to examine the engagement of supermarket staff in a health promotion project from a practice-oriented perspective.

**Methods:**

The study was based on qualitative data collected in the supermarket setting of Project SoL; a community-based health promotion project in Denmark. We conducted 26 in-depth interviews with store managers and other key staff members in seven participating supermarkets. In addition, we collected data on planning, implementation, and perceptions of supermarket staff of in-store interventions and other project-related activities. These field data included short telephone interviews, observational notes, photos, and audiotapes of meetings. Data were analysed from the perspective of practice theory.

**Results:**

Although supermarket staff found community-based health promotion meaningful to engage in, the study observed that their engagement was challenged by a business mindset, practical routines and structural requirements favouring sales promotion over health promotion. Nevertheless, there were also examples of how health promotion activities and ways of thinking were successfully incorporated in everyday staff practices during and after Project SoL.

**Conclusions:**

Our findings point to both potentials and challenges for using supermarkets as settings for health promotion. The voluntary engagement of supermarket staff in community-based health projects cannot stand alone but should be supplemented by more long-lasting strategies and policies regulating this and other food environments. Context-sensitive and practice-oriented analyses in local food environments could inform such strategies and policies to make sure they target unwanted elements and practices and not just individual behavior.

**Supplementary Information:**

The online version contains supplementary material available at 10.1186/s12889-023-15501-5.

## Background

Food stores are interesting settings for studies on change and continuity in food provisioning practices as western consumers buy most of their food products in food stores such as supermarkets [1, 2]. Marketing studies using both laboratory and field study methods have shown how the price, place, promotion, and assortment of food products influence purchase [3–5]. Public health researchers see great potentials in working together with supermarket staff and management on health promotion aiming to make supermarket environments more supportive of healthy living. Hence, supermarkets have been pointed to as interesting settings for environmental and educational initiatives encouraging healthier shopping and eating habits [6–9].

Overall, there are some common features across the intervention studies conducted in supermarkets: (1) The main focus of many studies has been the intervention effects on sales and consumer awareness and knowledge [1, 2]; (2) Process evaluations have focused on intervention-specific aspects of implementation such as fidelity [10] but seldom on how interventions involve or are perceived by supermarket staff [11, 12]; (3) Interventions are sometimes underpinned by social cognitive or social marketing theories [1], but most often findings are not interpreted theoretically. Thus, theory-based research exploring supermarket staff involvement and contextual aspects of interventions is largely non-existing. These shortcomings are not only characteristic for supermarket-based intervention studies; in fact, process evaluations of health behaviour interventions in general tend to pay limited attention to context, process and theory [13].

In line with others we argue that public health intervention research could learn from social science approaches to change and evaluation [13], not least by applying theories that more specifically address the translation of social and structural factors into everyday practices, including health-related practices [14]. Practice theory offers relevant perspectives on agency, structure and change [15, 16] with a focus on practitioners engaged in everyday life practices rather than individual actors and with an emphasis on the material environment as a context for and an outcome of such practices [17–19].

This paper presents results from a qualitative study on the practices of supermarkets staff and managers involved in Project SoL (from the Danish Sundhed og Lokalsamfund / Health and Local Community); a Danish community-based health promotion project [20–22]. The data were analysed within a theoretical framework based on practice theory [16]. The aim was to examine engagement and perceptions of supermarket staff and managers, and to examine whether their involvement in a health promotion intervention complemented or conflicted with their everyday business practices.

## Methods

### Project SoL – a community-based health promotion project

The conceptual framework as well as the intervention design of Project SoL have previously been described in detail [20–22]. Published results from Project SoL so far include evaluations of health promotion interventions in supermarkets and intervention effects on BMI [23–26]. The SoL project had a total duration of four years, including a 19-month intervention period (2012–2014) and aimed at promoting healthy eating and physical activity habits among families with small children. Conceptually, it was based on the supersetting approach encompassing highly participatory principles for developing and implementing interventions [20].

Project SoL took place in three small communities on the Danish island of Bornholm characterized by many low-income residents and health indicators below the regional average [27]. All supermarkets located in the three intervention communities were invited to join Project SoL as they were considered important community partners with potentials to make positive contributions to community health together with other local community stakeholders, e.g., child-care centers and local media. Seven local supermarkets of different size and from three different food retail groups accepted the invitation to participate in Project SoL and comprised the supermarket intervention on Bornholm (see Table 1).

Supermarket store managers and key staff members were involved in intervention development, implementation and to some extent evaluation in iterative processes throughout the project. Involvement included an initial community kick-off meeting, workshops for supermarkets, local action group meetings and regular meetings between supermarket staff and researchers. The supermarket intervention included environmental activities, such as placing and promoting healthy products in prominent store locations, and health-educational activities targeting children and their families, such as healthy treasure hunts, packed lunch workshops and community events with healthy food inspiration.

**Table 1:** Characteristics of participating stores

Store Name (no.)	Food retail group Name (no.)	Store type*	Number of staff members**
**SuperBrugsen (1)**	Coop (1)	Conventional supermarket, chain-owned	20
**SuperBrugsen (2)**	Coop (1)		20
**Kvickly (3)**	Coop (1)		70
**Dagligbrugsen (4)**	Coop (1)		8
**Netto (5)**	Dansk Supermarked (2)	Conventional/discount supermarket, chain-owned	15
**Netto (6)**	Dansk Supermarked (2)		17
**SPAR (7)**	SPAR (3)	Conventional supermarket, independent	6

*Based on the definitions by Corinna Hawkes (2008), but with the addition of a store type combining the conventional chain-owned supermarket with features from discount stores.

** Approximate number of staff members as reported by store managers. Number varies according to season and holidays. Numbers include full and part time staff.

### A practice-theoretical perspective on change and continuity

Practice theory [15, 16] was used to examine how supermarket staff perceived and engaged in the development and implementation of project activities. We conceived the supermarket involvement in Project SoL as an expression of practitioners (supermarket staff) carrying out social practices (intervention-related activities). The analysis primarily followed the ‘slim-line’ approach to practice theory proposed by Elizabeth Shove and colleagues [16, 19, 28–31]. This approach conceives social practices as results of on-going integration between three conceptual elements, which are interdependent, broad, and overlapping categories. The element of *meanings* emphasizes the normative, emotional and cultural beliefs and orientations shared by practitioners [16]. *Competencies* cover the practical and embodied routines, know-how, experiences, formal and informal knowledge and skills of carrying out practices [16], while *materials* includes materials and equipment that practitioners use, consume or interact with [16] as well as technologies, architecture and the material arrangements and environment that practices comprise and take place within [18, 28, 31].

*Change* and *continuity* are important aspects of practice theory as well. Practices are sustained when individual practitioners integrate or link elements through their daily performances. Practices are innovated or renewed when new or existing elements are linked in new ways. Practices are ended when elements are no longer actively linked. Hence, we examined the meanings, competences, and materials of selected intervention practices in supermarkets during Project SoL and how they were integrated or disintegrated by supermarket staff. Our primary access to examining these practice elements was through the performances—the doings (e.g. bodily activities) and sayings (e.g., speech) [15]—of supermarket staff and managers.

### Data collection

The data were collected from spring 2012 to spring 2014 at regular visits to the intervention communities. The rich and iterative data collection including dynamic sampling is characteristic for participatory intervention research [20, 32] and aimed to explore store practices at different times throughout the intervention. An overview of the collected qualitative data is provided in Table 2. First author LLW conducted all the interviews.

#### Interviews

The interview sample consisted of key staff members from the seven intervention stores and three head-offices representatives involved in Project Sol. When the stores joined Project SoL the store managers were made aware that researchers were interested in interviewing them and other staff members involved in interventions as part of the project documentation and research. Staff members were recruited during store visits, while head-office representatives were recruited by telephone-based requests. We initially recruited key staff members involved in the planning and implementation phases for baseline interviews (four store managers and the island-based regional sales manager from retail group 2) Three additional store managers and four section managers were recruited for and completed their first interview within the second project year (midterm) as we found that they played important roles in the project as it progressed. All participants were interviewed again by the end of the project, Two section managers were no longer employed in the supermarkets and the new section managers were interviewed instead. In addition, the regional sales manager and one director from retail group 1 and a director from retail group 2 were interviewed at the end of the project as we wanted to supplement our local data with perspectives from head-office representatives. Interviews with staff members were conducted in the staff room of supermarkets during working hours, while head-office representatives were interviewed at head office and over the telephone.

All in all, this resulted in 26 in-depth interviews (25–100 min). The interview guides (examples provided in additional files 1 and 2) were theoretically inspired by earlier versions of practice theory e.g., [33], life-world analysis [34] and themes from other interviews with supermarket staff e.g. [8, 11]. They were adapted to the professional function of the participant and the time of interview (baseline, midterm, or post-intervention), but all interviews included questions on a typical workday, considerations of their role in health promotion as well as expectations or experiences in relation to Project SoL. Hence, the specific practice-theoretical approach used to analyse data was not in place when we developed the interview guides. We do not see this as a challenge as our theoretical perspectives were compatible and since practice theory literature in general is unspecific when it comes to making analytical translations between theory and empirical data methods [35]. Moreover, as Braun and Clarke [36] note, it is important to distinguish between interview questions and the analytical questions guiding the coding and analysis of data, In the latter we have consistently used practice theory.

#### Observations and other qualitative data

In addition, we collected data on planning, implementation and supermarket staff perceptions of in-store interventions and other project-related activities throughout the project, including short telephone interviews, audiotapes of meetings, photographs, observational logbook notes, and implementation reports (inspired by e.g., Gittelsohn [37]). These field data were collected throughout the intervention period (the stores were visited at least once a month by either the first author or the local project coordinator). The data were collected for multiple purposes: (1) to supplement the self-reported performance data (formal interviews) with situated and ‘material’ examples of how project intentions were or were not enacted in practice. (2) to inform interview guides for the mid-term and post-interviews as they enabled us to ask very specific retrospective questions on implementation and practices and (3) to contribute with documentation of the supermarket intervention in other Project SoL publications.

**Table 2:** Overview of qualitative data collected

Activity	Participants	No. (time)	Duration (min)	Stores Represented	
Initial workshops	Store managers, key staff, researchers	Three workshops (baseline)	~ 60–90	All initial SoL stores, one workshop per chain	
Semi-structured interviews	Store Managers	14 interviews: N = 4 (baseline), N = 3 (mid-term) and N = 7 (post)	~ 30–90	All SoL stores	
	Section managers/ key staff	Eight interviews: Mid-term (N = 4) and post (N = 4)	~ 25–70	Store 1, 2, 3, 4, 6	
	Head office representatives	Five interviews Baseline (N = 1) and post (N = 4)	~ 40–60	Retail Group 1 and 2	
Other field data (e.g. observational notes, minutes, pictures)		Throughout the intervention period		All SoL stores	

### Data analysis

Interviews, workshops, and selected meetings were recorded on a digital voice recorder. Interviews were transcribed verbatim, whereas recordings from workshops, store visits and meetings were listened to while taking extensive notes. These meeting notes and the main author’s descriptive observational logbook notes were included in the analysis, while implementation reports and photographs were not coded but were used to inform interview guides and analysis. To ensure anonymity of participants the study does not give any information on their names and affiliations to chain and community. Extracts used in this paper were translated from Danish to British English.

We analysed our interview and field data through continuous processes of data familiarization, coding and theme-making across our different data using a theory-driven thematic analysis [36]. Initially transcripts and notes were read and re-read in full and initial analytical and deductive coding was done. Deductive codes included meanings, competencies, materials, change and continuity following key concepts from practice theory. After going back and forth between theory and data, the codes were collated into themes and named, while maintaining the deductive codes as part of the headlines (see additional file 3). QSR International’s NVivo 10 software was used to organize and analyse data. Initial coding and interpretation of data was done by LLW and subsequently reviewed, discussed and adjusted by all authors to ensure a broad, critical and reflexive reading [38].

## Results

Our findings on the engagement of supermarket staff are structured in three sections: (a) healthy placement and promotion practices; (b) health-educational practices; and (c) change, continuity, and sustainability of practices. The first two sections (a and b) are structured around the three themes arising when collating our theoretical codes: meanings, competencies, and materials, while section c assembles continuity and change codes across project practices into two themes. A table overview of themes can be found as additional file (see Additional file 3).

### Healthy placement and promotion practices

The collaboration with participating stores on promoting healthy food products was regarded as an essential and interesting aspect of Project SoL by the researchers. This first part of the analysis presents the supermarket staff enactment of placement and promotion practices (category a) during Project SoL. The three themes identified illustrate meanings, competencies, and materials of these practices (see Additional file 3).

#### Theme: healthy food choice as an individual responsibility (illustrating meanings)

The decision of the seven supermarkets to participate in Project SoL was primarily made by managers and directors at chain or company level, who explained that the chains mainly enrolled in the project to gain positive attention from local media and consumers. Thus, from a corporate perspective Project SoL was seen as a minor local experiment in good accordance with Corporate Social Responsibility (CSR) policies and local branding ambitions.

At store level the participation in Project SoL was seen as a daily store requirement in line with others, but supermarket staff also found the project aims sympathetic and relevant. At the same time, supermarket staff and managers were sceptical towards how local consumers would receive the healthy initiatives, not least changes in the store, as many of them were perceived to be price conscious and habitual in their food-shopping patterns:

*“We can try to display it in the store, and we really want to sell it, you know, but it also requires that somebody buys it”* (section manager, mid-term interview)

Thus, supermarket staff assigned the main responsibility for healthy living on the individual consumer and in their opinion, consumers were already provided with plenty of opportunities to make healthy food choices in their store. Still, promoting fruit and vegetables made sense to supermarket staff as the fruit and vegetable category could yield large revenues if promoted and placed right.

During the first three project months, many sales promotion ideas discussed by supermarket staff and researchers at baseline workshops were implemented. The change in the two discount stores (store 5 and 6/Netto) was especially noticeable as the stores placed large displays of fruit, vegetables, and whole-grain products in the front area of the store which was used to promote sugary snacks, flowers, or garden equipment before Project SoL. In addition, store 5 removed the VAT on fruit and vegetables for a three-month period supported by an enthusiastic director and regional sales manager. This attracted the attention of consumers and the local media. It also meant that managers from the competing stores were very close to withdrawing from the project in protest as they found that Netto broke the “rules of the project” by introducing price reductions and competition between the stores that were not sustainable in the long run. On the other hand, promoting fish and wholegrain products was not very appealing to supermarket staff. Fish was embedded in historical, social, and cultural meanings, skills, and infrastructure. There was no tradition of buying fish in the supermarket and the quality of supermarket fish was contested even by supermarket staff. They were sceptical about selling wholegrain products as well, referring to consumer preferences for non-wholegrain products. The problem of investing space and resources in promoting specific healthy products at the expense of other easier-to-sell less-healthy products, was pinpointed by one of the store managers:

*“I have to make some money for [retail group 1] and it doesn’t matter whether I make them by selling wholegrain or potato chips, because it is the bottom line I’m getting patted on the back for.”* (store manager, mid-term interview)

Despite the above-mentioned reservations, several interventions promoting wholegrain products and canned fish were implemented. Thus, to begin with the positive meanings ascribed to Project SoL were enough to secure project enactment.

#### Theme: incorporating new priorities in everyday practices (illustrating competencies)

Activities of the supermarket intervention were intended to be integrated in the normal store routines and hence rely on the skills and know-how of involved staff members. In general, supermarket staff had limited knowledge of health and nutrition and no experience of taking part in intervention projects. Their daily routines and ways of thinking evolved around promoting sales, not health, and most staff members were indifferent about the healthiness of products sold. At workshops and meetings with researchers they shared their know-how on how to influence the content of customer’s shopping bag:

*“Even if the grocery list says potato chips you can easily get them to choose carrots instead if it’s the first thing you see and you make a nice display and the price is ok”* (store manager,, initial workshop)

Thus, the skills needed in SoL–to successfully promote fruit, vegetables, fish, and wholegrain products—were overall not different from the skills of normal store routines. In intervention planning workshops, supermarket staff shared their practice-based know-how on sales promotion (best store locations, the local customer base, etc.) and researchers shared their knowledge from the literature (nutrition, intervention strategies, etc.) and together the groups generated store-specific interventions. However, creativity and new routines were also needed to promote healthy products. The section managers of fruit and vegetables had to devote more time on planning, ordering, displaying, and restocking than usual. To make eye-catching displays and place displays strategically required more creativity and coordination across staff members, but became a part of everyday routines very fast, according to one of the section managers:

*“To begin with we spoke of it a lot but now it’s kind of part of everyday life. [..] It’s just a part of everyday life that there must be healthy products in store in the most prominent place”* (section manager, mid-term interview)

Hence, in some stores the new routines had become normal, embedded routines within the first few months of the project. Using the practice-theoretical vocabulary, you could say that existing competencies were updated and used in new ways which implicated that health priorities now played a role in the daily placement and promotion practices.

#### Theme: non-selling wholegrain shelves, perishing fruits, and winner trophies (illustrating materials)

All stores already had an abundance of healthy products, but supermarket staff were unsure whether they could maintain a focus on fruit, vegetables, fish, and wholegrain products over a two-year project.

The stores were not exempted from following space management policies (e.g., the placement and number of facings of products in certain shelves following agreements between the chain and the manufacturers) and other chain restrictions (e.g., price campaigns) during Project SoL, hence there were chain policy constraints on which interventions to test and which products to promote. Nevertheless, participants pointed to the many remaining opportunities for in-store marketing of healthy products:

*We are of course ‘spaced’*^1^* on all our shelves, but on our tables, we can do exactly what we want. So, if we want to prioritize to really focus on whole-grain products in the store then we can dedicate a table or two”* (regional sales manager, initial workshop)

Table displays, endcaps and island bin displays were exempted from space management policies and the carrying out of placement and promotion practices relied on these physical objects.

The placement and promotion activities required both familiar materials (food products, displays), new materials (project posters, labels, recipes) and in some cases new use of the store infrastructure. Limited space, coolers and displays restrained project efforts to promote fruit and vegetables. Interventions promoting fish had to focus on canned fish products due to storage requirements.

After the first months of the project, the promotional activities decreased or attenuated in most stores. Staff members said this happened because of practical issues: fruit and vegetables perishing when promoted outside the cooled department, a need to reduce promotional activity in low-demand seasons and displays physically disturbing store and consumer routines:

*”People have to be able to move around with their walkers and (laughs).. so there’s no use in placing it at the middle of the aisle because that.. that wouldn’t work”* (section manager, mid-term interview)

However, infrastructure was only part of the reason. Lack of corporate support to invest sufficient time and space was also pointed to, not least by discount store managers. In the discount stores the numerous displays at the store entrance with fruit, vegetables and canned fish were suddenly substituted with cookies and other low-prized confectionery items. The store managers explained that changes were a consequence of a mandatory chain strategy initiated by the newly assigned sales director. Although the managers were embarrassed about not being able to maintain the healthy interventions they did not want to run any risks by opposing top-management decisions: *“If I don’t maintain my sales then I’ll lose my bonus. And I won’t give that up because of Project SoL”* (store manager, mid-term interview).

The success of the supermarket intervention was also challenged by a lack of staff engagement and implementation in the remaining stores (store 1,2, 3, 4 and 7). Most stores removed wholegrain products from prominent displays after a few days or weeks. Supermarket staff referred to space optimization and business logic when asked about these deviations from intervention plans:

*“When a section manager identifies a non-selling shelf.... then he’s not the type that just says: ‘it’s positive for SoL that we display it’ (laughs). No, they think in terms of money”* (store manager, post-intervention interview)

A ‘meal solution’ intervention in which the recipe and products for a healthy evening meal were placed in a centrally located cooler was unsuccessfully implemented as well. In one of the stores, the butcher proclaimed that he did not want to *“waste his coolers”* on promoting fish and vegetables, when he could promote high-profit meat products instead (meeting notes, spring 2013). The physical infrastructure represented loss of profit when not constantly optimized according to sale. Hence, a continued and dedicated promotion of healthy products was challenged by local infrastructure and physical characteristics of products (illustrating materials).

On the other hand, a sales competition activity between supermarkets was a successful attempt to strengthen supermarket staff engagement in placement and promotion activities. At a joint coordination meeting between researchers, the four store managers and the regional sales manager from retail group 1, researchers provided staff members with graphs on healthy product sales comparing project stores to control stores. Overall, the stores did not perform very well which troubled store managers so much that a three months ‘sales competition’ between the three stores focusing on promoting sales of vegetable roots, wholegrain breakfast products and canned fish products was agreed upon. A winner—the store selling most of the target product—was appointed each month by researchers based on sales data. Local childcare centres made trophies for the winning store and the intervention was covered by the local media. The intervention was well-implemented by supermarket staff, and all improved their sales of targeted healthy food products markedly. Thus, the perceived relevance and local media attention of activities (illustrating meanings) was renewed and boosted through the internal competition using sales figures and trophies (illustrating materials), which led to new enactments of healthy placement and promotion practices.

To sum up, the integration of the positive meanings ascribed to Project SoL (illustrating meanings), the updated promotional routines and skills of staff members (illustrating competencies) and the indifference with which products to sell (illustrating materials) enabled successful placement and promotion practices at times during the projects. However, the notion of individual consumer responsibility for health (illustrating meanings) and chain policies restricting products and local infrastructure (illustrating materials) were continuous challenges for the enactment of Project Sol-related practices in all stores.

### Health-educational practices in a supermarket setting

Project SoL also included health-educational activities in the store environment for children, families, and staff. As was the case with placement and promotion practices the enactment of health-educational practices varied across stores and during the project. This section presents three themes illustrating the three practice elements of health-educational practices (see Additional file 3).

#### Theme: local altruism and a long-term investment (illustrating meanings)

As mentioned, most supermarket staff were overall sympathetic to the idea of Project SoL. The project was seen as a chance for supermarkets to be part of a joint community effort hopefully promoting the health of community members while at the same time receiving positive media and consumer attention. Health-educational activities targeting children made immediate sense to all participants both morally and rationally. Children were considered to be susceptible and curious in relation to health education and thus an appropriate target group. This was important for supermarket staff fearing that an in-store focus on health targeting adult consumers could be interpreted as paternalistic and intrusive. As future consumers and as a possible link between stores and families, children were also considered interesting from a business point of view:

*“I love having children around in store because that creates a lively atmosphere. They are our future costumers as I always say. [..] if the children want to come here, then we get the parents to come here”* (section manager, post-intervention interview)

This extract is one of many examples of how business thinking and what can be called ‘local altruism’ were both present in supermarket staff practices. Staff members stressed the possible long-term effects of their engagement – for the community and for the store bottom-line. Moreover, perceived short-term effects of the activities included local media coverage, strengthened relations to parents, childcare centres and schools and goodwill from consumers for engaging in a good case. All this was believed to strengthen the store profile locally.

#### Theme: poor health interest and project ownership of staff members (illustrating competencies)

In all stores, staff were acquainted with most customers, but only stores from retailing group 1 had a tradition of hosting or being part of educational and community events. The skills and practical routines of health-educational practices were different from core store tasks such as stocking food. Thus, to carry out health-educational activities was challenging in some stores as it implicated disturbances in normal store practices, devotion of time and some collaborative, creative and communicative skills.

Hence, Project SoL staff and community partners were the main organizers of health-educational carried out in all stores’ activities, while activities with greater supermarket staff workload were less successfully rolled out. Examples of the former include a healthy treasure hunt and child-made in-store decorations. Examples of the latter include packed lunch workshops and in-store night events with healthy food promotions and activities, which were only implemented in stores with event experience and receiving chain support. Staff members who had experience in carrying out consumer events learned something as well, as it was new for them to take health into account in their event planning.

Other activities, such as guided healthy tours in supermarkets, were never carried out at all. Lack of health interest and knowledge of staff was pointed to by store managers as part of the reason. Moreover, throughout the project some supermarket participants continued to speak about Project SoL as *“your project”.* Taken together with the inconsistent implementation of placement and promotion activities this indicated that project ownership among some staff members was weak despite the participatory approach of the project.

To increase staff knowledge on health and to involve more staff members in Project SoL, hence, to strengthen competencies and meanings of SoL activities, all stores were offered a free two-day staff course. Despite several attempts, the intended course was never held as only few staff members signed up. Instead, two shorter educational events were held with staff members from stores 5, 6 and 7 focusing on product labelling and healthy hands-on tips. In the very positive course evaluations, participants mainly focused on how they could personally use the health information, not how it could be incorporated in their daily store tasks and benefit customers.

#### Theme: from marketplace to learning space (illustrating materials)

Project SoL led to new ways of using the store infrastructure. During the project, the store environment was not only a marketplace but also a learning space in which children learned about, drew, photographed, and tasted some of the healthy food products surrounding them. Stores were decorated with SoL merchandise (e.g., posters and shelf-talkers) as well as drawings and art projects made by children from local childcare centres. The supermarket staff were very motivated by these tangible symbols of the SoL project which they believed increased the *c*onsumer awareness of the project and gave the stores positive attention.

Moreover, activities such as a healthy treasure hunt and lunch packs workshops filled store areas with active children. A store manager from one of the discount stores was surprised by the positive attention and interest that the healthy treasure hunt received:

Store manager: “*The children were crazy about it; it was put on Facebook (laughs). They were crazy, you know, the word was spread*

Interviewer: *yes*

Store Manager: *They liked that something happened”* (store manager, post-intervention interview)

To stores that had no prior history of using the store environment in untraditional ways, this positive experience was an eye-opener. For staff members with more event experience it was new to have evening store events where consumers were offered whole-grain pasta, soup, and health information instead of the usual alcohol and snacks.

These new ways of using the store infrastructure and new product focus in store events might seem like insignificant temporary small changes but were part of rethinking the target groups and traditions of store community events.

To sum up, the enactment of health-educational practices happened as links were made between local altruism and stronger relations to local families (meanings), new use of products and store infrastructure at events (materials) and staff members gaining new knowledge and using the store in new ways assisted by intervention staff (competencies). However, the staff members lack of skills and interest in these interventions meant that the competencies element was weakly linked and destabilised this practice formation.

### Change, continuity, and sustainability of health-promoting practices

In this final section, we will present our findings on whether and how Project SoL changed staff practices and whether practices promoting health can be sustained in a supermarket setting. Two themes are presented in this section (see Additional file 3).

#### Theme: Project SoL influenced ‘business as usual’ (illustrating change and continuity)

When evaluating Project SoL, most participants pointed to some intense periods of healthy product promotion during the project which had made healthy shopping in their store easier for consumers. They also pointed to in-store health-educational activities that might have increased health awareness among children and their families. However, in general the participation in Project SoL was not perceived by supermarket staff to have dramatically changed consumer behaviour or store sales. Some stated that it would have required more radical interventions than what was possible within existing store conditions. Others pointed to untapped potentials of the project due to poor project management and the initial difficulties of supermarket staff and researchers in finding common grounds. Most interview participants did not perceive SoL interventions to have left any permanent changes in the store environment:

Interviewer: *“Does the store in any way look different now from when we started the project?*

Store manager: *Not now, I have to say, because we’re back in the old way of doing things. It’s kind of easier to do what you are used to doing”* (store manager, post-intervention interview)

Hence, it seemed as if changes had been temporary and that old practices had been continued (elements of meanings, competencies and materials were back in the configurations prevailing before the project started). However, changes in both store environment and performances were identified. For example, fruit and vegetables were permanently promoted in displays located in the store entrance area and in the non-food section in store 3. In store 2 the store manager, who earlier in the interview had more or less rejected effects of Project SoL, suddenly said in an aside:

*“I don’t place six or eight quarter pallets of chips at the store entrance any longer. We really haven’t done that since [project start], we stopped doing that [..]I’ve just decided that Coca-Cola is placed somewhere else now. It’s placed in the bottom of the store”* (store manager, post-intervention interview)

There were also many small-scale examples of how supermarket staff had incorporated health considerations in store practices, such as displaying wholegrain products alongside conventional bread and breakfast products and more frequently rejecting campaign offers from confectionery sellers dropping by. Some staff members also explained that the project had made an impact on their eating practices at home.

The SoL Project had also required supermarket staff to collaborate with researchers. In their everyday routines supermarket staff were used to top-down decision-making and short-term deadlines and had perceived the participatory and iterative approach of Project SoL with some scepticism. Furthermore, some staff members felt estranged to the way researchers communicated and worked. Especially the head researchers were perceived with wonder:

*“They live in a completely different world!”* [..] *“Professors sitting reading books and dissertations... it’s just not for me which is why I did not choose a job like that”* (store manager, baseline interview)

Hence, the *perception of a gap between supermarket staff and researchers* was expressed by some participants at the start of the project, but this gap seemed to decrease as the project and the collaboration progressed. Thus, the collaboration with researchers was called fun, interesting and a pleasant break from usual work routines after initial start-up challenges.

#### Theme: sustaining healthy supermarket practices (illustrating sustainability)

One attempt to anchor SoL activities locally was through local collaboration. Local action groups were set up during the last part of the project to increase coordination and synergy between community settings and to create a foundation for making community activities that were sustainable when the official part of Project SoL ended. Three of the stores (store 2, 3, 4) were represented in the local action groups and carried out successful community events in collaboration with commerce associations and other community actors. They wanted to continue their local engagement but questioned whether the groups would continue without the administrative support from the local project coordinator. The remaining stores were not represented in these groups as staff members were unable to attend meetings during working hours and unwilling to attend meetings in their leisure time. Moreover, they felt increasingly challenged by chain concepts, additional opening hours and reduced staff budgets. Hence, they admitted feeling relieved when the project finally ended. As elaborated by one of the discount store managers:

*“There’s no time for it, there’s no room for it. We don’t have the possibilities [..]*

*If we could decide ourselves; then yes - then it would be fine. Then we could surely have done a lot of other things. But when we need to follow the chain concept; then no”* (store manager, post interview)

While local supermarket staff in this way pointed to the importance of corporate support, e.g., additional resources and more flexible chain regulations, head-office representatives pointed to opportunities and barriers for health promotion outside the supermarket. They stressed how health was an individual and a political responsibility:

*” Sure, we can do a lot within food retailing, but it’s not just us [..] we can place some things differently but there are some much wider and bigger things at stake”* (director, post interview).

Thus, supermarket staff at all levels seemed to allocate the primary responsibility for health elsewhere. Still, some of the involved staff seemed more conscious about their potential role in community health and admitted thinking differently about their health responsibility than when they were asked at baseline.

## Discussion

Drawing on qualitative data from a two-year-long field work conducted within a Danish community-based health promotion project and interpreted within a practice-theoretical framework, we have presented examples of intervention-related practices of supermarket staff. The store activity varied during the intervention period and some of the practices were rather successfully integrated in daily store routines, while others were not.

We will now discuss our findings in relation to findings in the literature, reflect on the insights and implications of using a practice-oriented perspective and point to some overall strengths and limitations of our study.

### Responsibility for consumer and community health

Despite supermarket staff and representatives’ overall sympathy with Project SoL, we found that they often placed responsibility for health with other actors, not least the individual consumer, or placed it in other practices outside the supermarket. From their perspective, consumers were already provided with plenty of opportunities to make healthy food choices. However, they did not seem to reflect on the balance between the amount of healthy versus unhealthy food items and the promotion of these foods in a supermarket. For example, studies have found that energy-dense snacks and sugary beverages take up more shelf space than fruit and vegetables within all store types [39, 40] and that price promotions include fatty and sugary foods twice as much as fruits and vegetables [41]. Our findings are somewhat in contrast to the findings of Middel and colleagues who in a systematic review from 2019 concludes that most retailers showed responsibility and awareness of the health of their community [42]. The intervention stores in the present project had not previously participated in health promotion, which may explain some of the discrepancy.

On the other hand and more similar to our findings, the literature on Corporate Social Responsibility has described how the ‘macro-level’ corporate aspirations, strategies and reports on health are only poorly reflected in the everyday operations of ‘micro-level’ food retailers in a competitive business environment [43, 44]. Moreover, in practice supermarket’s healthy initiatives tend to defer the corporate responsibility for health and place it on individual consumers [45]. Likewise, we identified some discrepancies between the head-office enrolment in the project and underlying practical and structural conditions making health promotion very difficult for local staff members to enact in practice. Hence, the corporate willingness to truly take on responsibility for consumer health might be questioned.

### Lessons learned from store-based health-promotion interventions

Despite the un-traditional theoretical approach taken in this paper, many of the findings and experiences reported are in line with the lessons learned from working with staff in store-based health promotion interventions described in the international literature. For example, we found that limited space, coolers, time, staff resources, perceived low customer demand, and the priority of promoting sales challenged the linking of elements into sustainable health-promoting supermarket staff practices. This is in line with studies describing financial and practical issues as barriers to health promotion in food stores [10, 11, 42, 46–49]. However, although bottom-line thinking sometimes hindered or dis-integrated intervention practices, our work also identified examples of how the business orientation of supermarket staff walked hand in hand with health promotion. For example, the project engagement of store members was renewed because of friendly intra- and inter-chain competition on promoting healthy products. Examples of friendly competition were also observed in two other studies, even though this was more formal, in the form of retailer associations [33, 38].

Moreover in line with other store-based intervention studies, we found that business and consumer orientation needs to be incorporated in interventions [42, 48, 50, 51], that building a trustful relationship between researchers and store managers takes time [48, 52], is challenged by unclear communication [10] and staff time [49] and that the involvement of head-office representatives from the supermarket groups are important to engage causal local staff members [52]. Moreover these findings seem to be transferable across countries and store size, as most of the supermarket-based intervention studies take place in smaller convenience and corner stores in USA and United Kingdom, while the SoL stores were all supermarkets (albeit small) following the definition of Corinna Hawkes [53] and located in a geographically isolated area of Denmark.

### Insights and implications of using a practice-theoretical perspective

Our practice-theoretical perspective also provides new insights on using supermarkets as a setting for health promotion than the existing literature. By conceiving supermarket staff as practitioners rather than as implementers and by conceiving supermarkets as settings for everyday work practices rather than just intervention settings, we began to understand staff and management engagement in the project as results of practical involvement and social interactions responsible for linking or de-linking meanings, competencies, and materials rather than as results of individual motivation. For example, our work points to local media coverage and strengthened relations to consumers and other community members as meaningful aspects and outcomes of supermarket staff involvement in community-based health promotion. As mentioned in relation to the theme ‘Local altruism and long-term investment’, supermarket staff saw the project as a chance to be part of a joint community effort. Furthermore, the interaction between supermarket staff and researchers was also an important aspect of Project SoL. Social interaction is not adequately addressed in practice theory according to some scholars [54, 55]. The gap between supermarket staff and researchers narrowed as staff and researchers got to know each other and carried out activities together. Hence, through shared practice-based experience and the positive social meanings ascribed to in-store health promotion activities and Project SoL, supermarket staff and researchers became more open towards incorporating each other’s knowledge and know-how in project-related practices [16]. Hence, elements of meanings and competencies were linked in new ways which was important for the enactment of project practices.

These examples show that a practice-oriented perspective offers a different take on supermarket staff involvement than many of the studies referred to above focusing on the motivation of the individual store manager [10, 11, 51, 56] e.g., studies treating individual staff motivation as a predefined determinant of intervention outcomes e.g. [11] or focusing on barriers and drivers to behaviour change, e.g. studies looking at the effects of financial incentives [51]. Instead of conceiving corporate or policy regulations as external factors influencing individual behaviours, practice theorists like Shove and her followers see such aspects as embedded in, and part of, practice [19, 31, 57]. As Shove explains: “we consider methods of planning and policy-making as practices in their own right, and as arrangements that are part of rather than outside the ongoing flux of daily life.” [31]. In our analysis, the space management policies and bonus schemes of supermarket chains were mainly described in relation to the materials elements as they played a role for the use and allocation of products and store infrastructure. Rather than external barriers or drivers, these corporate policies can be seen as ‘material arrangements’ or ‘co-existing forms of materiality’ that exist in the ‘background’ of local supermarket practices [30, 31] linking elements and practices at different levels [18, 28], e.g., micro- and macro-practices of food retailers.

At first glance, Project SoL did not seem to foster much change in the practices of involved supermarket staff as most of the healthy interventions were only implemented for a short while (cf. theme Project SoL influenced ‘business as usual’). However, our study did identify some more permanent changes in staff doings and sayings indicating opportunities for health promotion, e.g., more awareness on promoting healthy products and taking more responsibility for community health and for healthy eating at home. Such findings mirror the observations of Hargreaves [54] in a practice-theoretical analysis of a pro-environmental behaviour change initiative. The initiative did not lead to any obvious and radical changes in everyday working life but by using observations and interviews Hargreaves found examples of subtle shifts towards more pro-environmental working practices [54]. We believe that our practice-theoretical perspective made us more attentive to such minor changes in everyday practices, as well as other contextual aspects of relevance to the intervention, that is generally not identified in process evaluations of store-based interventions focusing on dose, reach and fidelity measures [37, 49, 58–60].

### Strengths and limitations

Practice theory does not give specific instructions to the appropriate qualitative design. In our reporting and evaluation of the qualitative methods employed, we were inspired by the EPICURE agenda [38]. This agenda suggests researchers to evaluate qualitative research using the seven items (engagement, processing, interpretation, critique, usefulness, relevance and ethics) in an “reflexive dialogue” [38]. One of the main strengths of this study relates to *engagement*. The collaboration between supermarket staff and researchers for more than a two-year long period gave us access to rich data and enabled us to describe some specific mundane practical dilemmas as well as some difficult overall tightropes characterizing the work with health promotion in the Bornholm supermarket setting. The prolonged stay also had implications for the *processing* and *interpretation* of data, as we were able to flexibly adjust research questions and theoretical perspectives, to collect additional data when needed and to make a context-sensitive interpretation of our data. The originality of our study also lies in the way theory was operationalized and applied to data from a real-life supermarket intervention setting.

Limitations of our study include *engagement, processing*, and *interpretation* aspects as well as *ethics*. Our active engagement in the field can also be seen as a limitation. The first author and main interviewer of this paper had intertwined agendas and roles. She engaged in the field to assess, document, and contribute to the development of intervention activities, while at the same time examining and analyzing such events. This might have led to some social desirability bias, e.g., participants expressing more positive attitudes towards health and Project SoL. The dual role might also have played a role for the processing and interpretation of data. For example, as staff members and researcher got to know each other better, staff members sometimes shared interesting but sensitive information that we found unethical to publish. Such dilemmas are frequently described in participatory research and field work [61, 62] and was mitigated by being transparent and respectful.

Although our data comprise of different data collected in our long presence in the field, our analysis has primarily relied on interviews and other verbal accounts. Some authors point to interviews as giving limited knowledge on practical and embodied activity [63], while others are more positive on the use of interviews as valid sources to practice knowledge [35]. We are also aware that our practice-theoretical perspective has influenced and sometimes limited our interpretation and presentation of events. The interdependence between elements and between elements, practices, and ‘context’, e.g., material arrangements, challenged the analysis and the presentation of data, as concepts were sometimes hard to distinguish from each other. Such methodological issues have been discussed by other researchers using practice theory as well e.g. [35]. Moreover, the analytical focus on elements and practices shared across the data has implicated less attention to differences within stores and between the involved stores and communities, although we have pointed to some differences, e.g. temporal changes in activity level and corporate support.

Finally, a major limitation of the study design in respect to its *usefulness* and *scientific relevance* for public health is the narrow focus on supermarkets. We acknowledge that effective community-based health promotion require interventions targeting other parts of the local food environment as well [64–68].

## Conclusions

We conclude that sales promotion was favoured over health promotion in the daily store practices even during a health promotion project. This is not a surprising conclusion, but our analysis has pointed to some nuances and mechanisms of supermarket staff involvement in health promotion. The challenges connected to using the supermarket as a setting for health promotion do not just origin in the motivation or demotivation of individual supermarket staff members or constraining external factors. Rather supermarket staff are part of a complex bundle of actors, practices and systems promoting consumption rather than healthy diets. Hence, the most interesting part of our study is not what the project *did not change* in supermarket staff practices, but rather the little alterations that *did innovate* some business practices during the project and left some healthy marks behind.

The voluntary engagement of supermarket actors in community-based health projects cannot stand alone but should be supplemented with more long-lasting strategies and policies regulating the food retailing industry and related global and local food actors and environments. The literature on obesogenic environments has addressed environmental contributors to obesity and pointed to potentially potent strategies, such as taxes on sugary beverages and restrictions on food marketing. Context-sensitive and practice-oriented analyses in local food environments could inform such strategies and policies to make sure they target unwanted elements and practices and not just individual behavior of consumers and retailers.

## Electronic supplementary material

Below is the link to the electronic supplementary material.

Supplementary Material 1

Supplementary Material 2

Supplementary Material 3

## Data Availability

The interview data used in this study are not publicly available as consent from study participants to access and use the data was restricted to analyses and publications within Project SoL. The data are available on request from the corresponding author.

## Footnotes

1 Even in the original Danish quote the sales manager used the word ”spaced”, using the British word space management as a Danish verb. Hence, “spaced in all our shelves” means that all shelves should be stocked according to a master plan provided by the chain following agreements with manufacturers. All local supermarket managers in our study used this expression.
