# Supplementary material for: Involving supermarkets in health promotion interventions in the Danish Project SoL. A practice-oriented qualitative study on the engagement of supermarket staff and managers
Source: BMC Public Health. 2023 Apr 18;23:706. doi: 10.1186/s12889-023-15501-5 (PMC10111755; doi:10.1186/s12889-023-15501-5)
Supplement: Supplementary file 1 — Supplementary Material 1 [file 12889_2023_15501_MOESM1_ESM.pdf]

Interview guide store managers baseline generic

<u>Briefing</u>

- The aim of the interview
- Baseline and again at project completion, anonymity to the extent possible

<u>A little about the store:</u>

- The store size: number and type of staff, square meters
- The store in relation to neighboring stores (Is there a "division of roles"? Do customers buy, for example, a smaller proportion of the product quantity at your store?)
- Turnover and profits in light of the declining population figure in Bornholm, fewer tourists, financial crisis, competition, etc.?
- The customer group and shopping patterns (health, demography, etc.)

<u>A little about the store itself:</u>

- How long have you been in store x and chain x?
- Is Bornholm different from the other places you have worked (if previously employed elsewhere)?
- Why did you choose to train as a retail assistant?
- How is your time as a store manager divided – administration/finance, in the store, management?
- How are you remunerated as a store manager – not the amount but the method of remuneration – fixed salary, commission, bonus… (Does the store manager have anything at stake financially?). Are you assessed on other things than sales (strategies, campaigns, CSR…)?
- What do you understand by health?
- Do you perceive yourself as knowledgeable about healthy diet, dietary advice, health recommendations, etc.?

<u>A little about the chain and store from a health perspective</u>

- Do you feel that your chain wishes to distinguish itself particularly within the health agenda, or are other agendas more important (organic food, fairtrade, price)? Has this changed during the last couple of years?
- Is there a management focus on health and social responsibility? Are courses offered to managers and staff? Are there policies on healthy product assortment and product placements?
- What do you think about the store's provision of healthy products and healthy initiatives in comparison with its competitors on the island?
- In surveys, consumers often say that health is an important criterion for product choice and that they would like the store to help them make healthier choices. Is that also your experience? What response do you get from your customers about the store, products, initiatives (price, health, organic food, etc.)?

<u>Project SoL – Health and Local Community</u>

Chronology and the store's resources

- When did you first hear about the project? Can you remember what you thought?
- When did you decide to join the project – and why? Who made the decision? What did the decision require (green light from management, etc.)?
- What do you hope to get out of the project?
- Have you participated in anything similar before?

Interview guide store managers baseline generic

- Are your staff interested in health and local projects/initiatives besides the normal day-to-day running of the store? How do you assess their resources and commitment in relation to SoL?
- What time and resources do you set aside for this project (staff resources, own resources, financial)?
- What resources were needed before your store was successfully able to meet the goals regarding better food and exercise habits for the island's families with small children?

The process so far

- How do you think the preliminary meetings have gone: preliminary meetings, kick-off meetings, meetings with the research group, meeting with the other supermarkets, etc.?
- What has gone well/less well in the collaboration?
- What potential challenges do you envision?
- Do you have suggestions for how the process can be optimized/improved?

Expectations and challenges

- What strengths do you see in the project?
- What challenges do you see in the project?
- Is it a problem that other chains are involved in the project? Does it promote collaboration and local commitment or competition? Confidentiality about sales and campaigns?
- What do you expect to come out of Project SoL? Ask open question first, after which further questions can be asked about sales, branding, media attention, local community participation, etc.
- Imagine that we are in March 2014 and Project SoL has just ended. What initiatives and effects do you hope to be able to look back on? (Possibly ask about the ideal scenario and the more realistic scenario)

Health, local community and grocery shopping

- The local grocery store is a social hub for the community. As store manager/district manager, do you see yourself as having a joint responsibility for the wellbeing and development of the local community?
- Who has responsibility for the individual's health – the individual, the family, the school, the politicians, store retailers? What responsibility do you have as a store for what your customers put in their shopping basket?
- In your experience, how can you most effectively promote the sale of healthy products? Have these strategies/methods been brought into play in SoL? Why/why not? What barriers are there to promoting the sale of healthy products – space management, spaces bought by certain suppliers/brands? Management decisions at chain level? Time? Finances?
- I will now mention various initiatives. Come with your immediate response to whether you can imagine introducing the initiatives in your store – not necessarily as part of Project SoL:

  Candy-free checkout?
  VAT exemption on fruit and vegetables?
  Allow employees time to organize and participate in health activities during working hours – e.g. health day together with the local daycare center?
  Give fizzy drinks and candy less visible placements in the store?
  Make employees health ambassadors?
  Sponsor a local youth football team or running team?

How far should the store go in pursuing the responsible agenda? How do you perceive the balance between financial bottom-line and the other bottom-lines?

Do you have anything to add?

Debriefing
