# Supplementary material for: Involving supermarkets in health promotion interventions in the Danish Project SoL. A practice-oriented qualitative study on the engagement of supermarket staff and managers
Source: BMC Public Health. 2023 Apr 18;23:706. doi: 10.1186/s12889-023-15501-5 (PMC10111755; doi:10.1186/s12889-023-15501-5)
Supplement: Supplementary file 2 — Supplementary Material 2 [file 12889_2023_15501_MOESM2_ESM.pdf]

Interview guide - head-office representatives post example

Briefing

- The aim of the interview and how data are used

A little about the store manager:

- Tell a little about your background and how you ended up in Retail group 1?
- Briefly tell about a typical day as information director, e.g. last Friday?
- What do you understand by health?
- Do you perceive yourself as being interested in and knowledgeable about healthy diet, dietary advice, health recommendations, etc.?

Project SoL – Health and Local Community

Chronology and process

- When did you first hear about Project SoL? Can you remember what you thought?
- If you had to explain to a Martian what Project SoL is, what would you say?
- Who was involved in the decision regarding Retail group 1's participation and what were the considerations behind the participation?
- What did Retail group 1 expect to get out of participating in the project?
- Were there some "ground rules" laid down for the participation – e.g. about which experiments/initiatives the participating stores were allowed to try, the head office's role and contributions to the project, etc.?
- How was the project participation communicated to the stores in the three local communities, and was it made compulsory for them to participate?
- Which meetings and activities have you been involved in connected with SoL? How have you experienced these?
- How have you been involved in the process and informed about the project along the way - by district managers, by store managers, by Project SoL..? Can you mention any specific matters you have been involved in or have heard about?
- How you have generally perceived the collaboration with the researchers in SoL? What has gone well/less in the collaboration? If possible, provide specific examples (information level, communication, planning, agreements, evaluation)
- Do you have suggestions for how the process could have been improved?

Project SoL and normal working day in the grocery store

- Is it your perception that Retail group 1's regional managers, sales managers and store managers are interested in health, social responsibility projects and local projects/initiatives besides the normal running operations?
- How have you generally experienced the involvement of regional managers (store managers/employees' specific involvement and interest in SoL)? What motivates them in their work (sales, media coverage, customer awareness) and is it compatible with a project like SoL?
- What are the barriers and possibilities for promoting the sale of healthy products and conducting health activities in the store (space management, management decisions at store, chain and corporate level, time, finance, store manager and employees' desire, interest and competencies) – in general and specifically in relation to SoL?
- What opportunities and challenges exist in connection with participating in a research project (documentation: logbook and camera, interviews, agreements on implementation..)

Interview guide - head-office representatives post example

- Is there management support for the store to work with health and social responsibility, both generally and in relation to SoL? How does this support manifest itself specifically? In what way do you support healthy initiatives in the stores themselves?

Success criteria, effort and outcomes

- What have Retail group 1 got out of participating in the project on Bornholm?
- Does anyone in Retail group 1 know about the project? Has it been a topic of discussion (internally, media stories)? Is the project interesting at chain or corporate level and communication-wise?
- Do you have any sense of Project SoL Bornholm's effect (in a broad sense)?
- Project SoL has some ambitious objectives, e.g. to increase sales of foods such as fruit & vegetables, wholegrain and fish by 15 percent. In your opinion, what is needed to achieve such a target?
- Which resources and circumstances would be needed for participating SoL stores to (more) successfully help achieve the goals of better food and exercise habits for the town's/island's young child families? Could you have given the stores greater freedom of action, salary funds, financial incentives, etc.? (Specific example: If a store manager phoned and asked for financial support for an event, payroll funds for a ”SoL manager”, to refrain from unhealthy displays or similar).
- Project SoL has been called Denmark's largest nudging project, and there has been great (local) media and research interest, particularly regarding the role of stores. In this light, do you think that the stores' and chain's efforts in SoL have been adequate? Could we in the project have done anything to increase commitment out in the stores? Could we have collaborated more with chain and head office on activities, store initiatives and media, and thereby seen greater results?

<u>Health and the grocery store's joint responsibility</u>

- How do you perceive the health agenda in Retail group 1 right now in relation to other responsibility agendas and bottom-line, etc.? Has this changed over the last couple of years with financial crisis, etc.?
- In surveys, consumers often say that health is an important criterion for their choice of products and that they wish the store to help them make healthier choices. Is this also your experience?
- Who has responsibility for the individual's health? What responsibility do you as a store/chain/corporation have for what your customers put in their shopping basket?
- Does the individual store manager, for example, have a joint responsibility for health and development in the local community – and do you feel that store managers live up to such a responsibility? Do you think that a project like SoL can contribute to promoting this or has changed this?
- In your opinion, when can a supermarket/corporation profile itself as undertaking a joint responsibility for improving public health?
- Which specific initiatives have you launched to support customers in making healthy choices, and what is your experience of the effect of this on sales and customer awareness?
- There are fewer initiatives that inhibit the sale of unhealthy products – e.g. candy-free checkout – and in the media you justify this partly on the basis of falling turnover. Does the financial bottom line always take precedence over what is responsible, so you only prioritize health as long as it is good business?
- How far can and will the corporation/chain/store go to promote the healthy agenda?

Do you have anything to add?

Debriefing
