# Supplementary material for: Involving supermarkets in health promotion interventions in the Danish Project SoL. A practice-oriented qualitative study on the engagement of supermarket staff and managers
Source: BMC Public Health. 2023 Apr 18;23:706. doi: 10.1186/s12889-023-15501-5 (PMC10111755; doi:10.1186/s12889-023-15501-5)
Supplement: Supplementary file 3 — Supplementary Material 3 [file 12889_2023_15501_MOESM3_ESM.pdf]

Overview of themes

	Healthy placement and promotion practices (a)	Health-educational practices in a supermarket setting (b)
Theoretical coding category		
Meanings	Theme: *Healthy food choice as an individual responsibility*	Theme: *Local altruism and a long-term investment*
Competencies	Theme: *Incorporating new priorities in everyday practices*	Theme: *Poor health interest and project ownership of staff members*
Materials	Theme: *Non-selling shelves, perishable fruits and winner trophies*	Theme: *From marketplace to learning space*
	Change, continuity, and sustainability of health-promoting practices (c)	
Change and continuity	Theme: *Project SoL influenced 'business as usual'*	
Sustainability	Theme: *Sustaining healthy supermarket practices*	
